# Pulmonary Embolism Complicating an Extended Postanesthesia Care Unit Stay: A Case Report

**DOI:** 10.7759/cureus.115203

**Published:** 2026-08-26

**Authors:** Mustafa Suliman, Umm E Amara, Umme Nashrah, Nissar Shaikh

**Affiliations:** 1 Medical Education, University of Science and Technology, Omdurman, SDN; 2 Obstetrics and Gynaecology, Deccan College of Medical Sciences, Hyderabad, IND; 3 Medicine, Deccan College of Medical Sciences, Hyderabad, IND; 4 Surgical Intensive Care, Hamad Medical Corporation, Doha, QAT

**Keywords:** deep venous thrombosis, enoxaparin, extended pacu stay, post-anesthesia care unit, postoperative complications, pulmonary embolism, venous thromboembolism

## Abstract

Extended postanesthesia care unit (PACU) stays, often required when high-dependency beds are unavailable, are generally safe but not free of complications. We report a 39-year-old man who underwent emergency laparotomy for a bleeding duodenal tumor with transfusion and vasopressor support, who remained in the PACU beyond the usual period. On postoperative day one, he developed tachycardia, tachypnea, and an unexplained hemoglobin drop. Computed tomography confirmed a right sub-segmental pulmonary embolism, and Doppler ultrasound revealed a left lower-limb deep venous thrombosis. He was treated with therapeutic enoxaparin and recovered fully. Clinicians should maintain a high index of suspicion for venous thromboembolism during extended PACU stays.

## Introduction

A routine postanesthesia care unit (PACU) stay typically lasts one to three hours, during which patients recover from anesthesia, are monitored for immediate postoperative complications, and are assessed for readiness for discharge to the ward [1]. An extended PACU stay describes patients remaining in the PACU or recovery room beyond this expected recovery window - most commonly for six hours or more, or overnight - typically because no ward, high-dependency unit (HDU), or intensive care unit (ICU) bed is available after prolonged or complex surgery or intraoperative instability, or in patients with multiple comorbidities [1]. While this practice can help patients avoid an unnecessary ICU admission when recovery is rapid, it is not without risk [2,3]. Extended PACU stays have been associated with cardiorespiratory instability, hypothermia, delirium, hospital-acquired infection, and immobility-related complications [4].

Deep venous thrombosis (DVT) and pulmonary embolism (PE) are well-recognized and not uncommon complications of the postoperative period across all care settings, including the surgical ward, HDU, and ICU. However, PE occurring specifically during an extended PACU stay - as a distinct care environment - has been rarely reported in the literature, with only one prior case described [5]. The extended PACU environment provides a level of monitoring comparable to intermediate-level or ICU care, and shares similar thromboembolic risk factors with other postoperative care settings. Surgical tissue trauma, activation of the coagulation cascade, general anesthesia, and PACU immobility likely combine to increase thromboembolic risk in this setting [6]. We describe a patient who developed DVT and PE during an extended PACU stay following emergency laparotomy for a bleeding duodenal tumor, to highlight this underrecognized complication and the importance of early suspicion and imaging.

## Case presentation

A 39-year-old previously healthy man presented to the emergency department with severe abdominal pain, hematochezia, and vomiting after referral from another hospital. On examination, the right upper quadrant was tender with guarding, and he was tachycardic (110-120 beats/min). Hemoglobin was 7.2 g/dL (Table 1). Cross-sectional imaging could not be performed preoperatively as the patient was unable to tolerate a supine position; upper gastrointestinal endoscopy was therefore pursued as the initial diagnostic investigation, which identified a bleeding duodenal mass. He underwent emergency exploratory laparotomy with excision of the bleeding tumor, requiring four units of packed red blood cells (pRBCs) and a noradrenaline infusion to maintain hemodynamic stability. Surgery lasted five hours; vasopressors were weaned, and he was extubated at its conclusion. Intermittent pneumatic compression devices were applied intraoperatively for mechanical venous thromboembolism (VTE) prophylaxis. Pharmacological VTE prophylaxis was not administered preoperatively or intraoperatively given the active bleeding and anticipated major surgery.

**Table 1:** Summary of laboratory values, vital signs, and clinical findings. POD: postoperative day; CT: computed tomography; DVT: deep venous thrombosis; PACU: postanesthesia care unit

Parameter	Value	Reference range	Timing
Admission (emergency department)			
Hemoglobin	7.2 g/dL	13.5-17.5 g/dL	On presentation
Heart rate	110-120 beats/min	60-100 beats/min	On presentation
Postoperative period			
Hemoglobin	11.3 g/dL	13.5-17.5 g/dL	Immediate postoperative period (POD 0)
Hemoglobin	7.1 g/dL	13.5-17.5 g/dL	POD 1
Heart rate	122-140 beats/min	60-100 beats/min	POD 1
Respiratory rate	22-24 breaths/min	12-20 breaths/min	POD 1
Oxygen therapy	High-flow nasal cannula, 50 L/min	Room air	POD 1
Diagnostic findings and treatment			
CT abdomen	No intra-abdominal bleeding	No active bleeding	POD 1
CT pulmonary angiography	Right sub-segmental pulmonary embolism	No filling defect	POD 1
Doppler ultrasound (lower limbs)	Acute DVT, left superficial femoral vein	No thrombus	POD 1
Thrombophilia work-up	Unremarkable	No thrombophilic disorder	During admission
Treatment			
Packed red blood cells	4 units	-	During surgery
Noradrenaline	Infusion (weaned post-op)	-	During surgery
Intermittent pneumatic compression	Applied	-	During surgery
Sequential compression devices	Continued in PACU	-	Postoperative period
Packed red blood cells	2 units	-	POD 1
Enoxaparin	70 mg (~1 mg/kg) twice daily (therapeutic)	Prophylactic: 40 mg once daily	From diagnosis to discharge
Outcome			
Heart rate	Normalized	60-100 beats/min	POD 3
Hemoglobin	Stabilized	13.5-17.5 g/dL	POD 3
Length of hospital stay	5 days	-	Discharge on day 5
Anticoagulation	Enoxaparin (therapeutic, 3-month course)	-	At discharge

**Figure 1:**
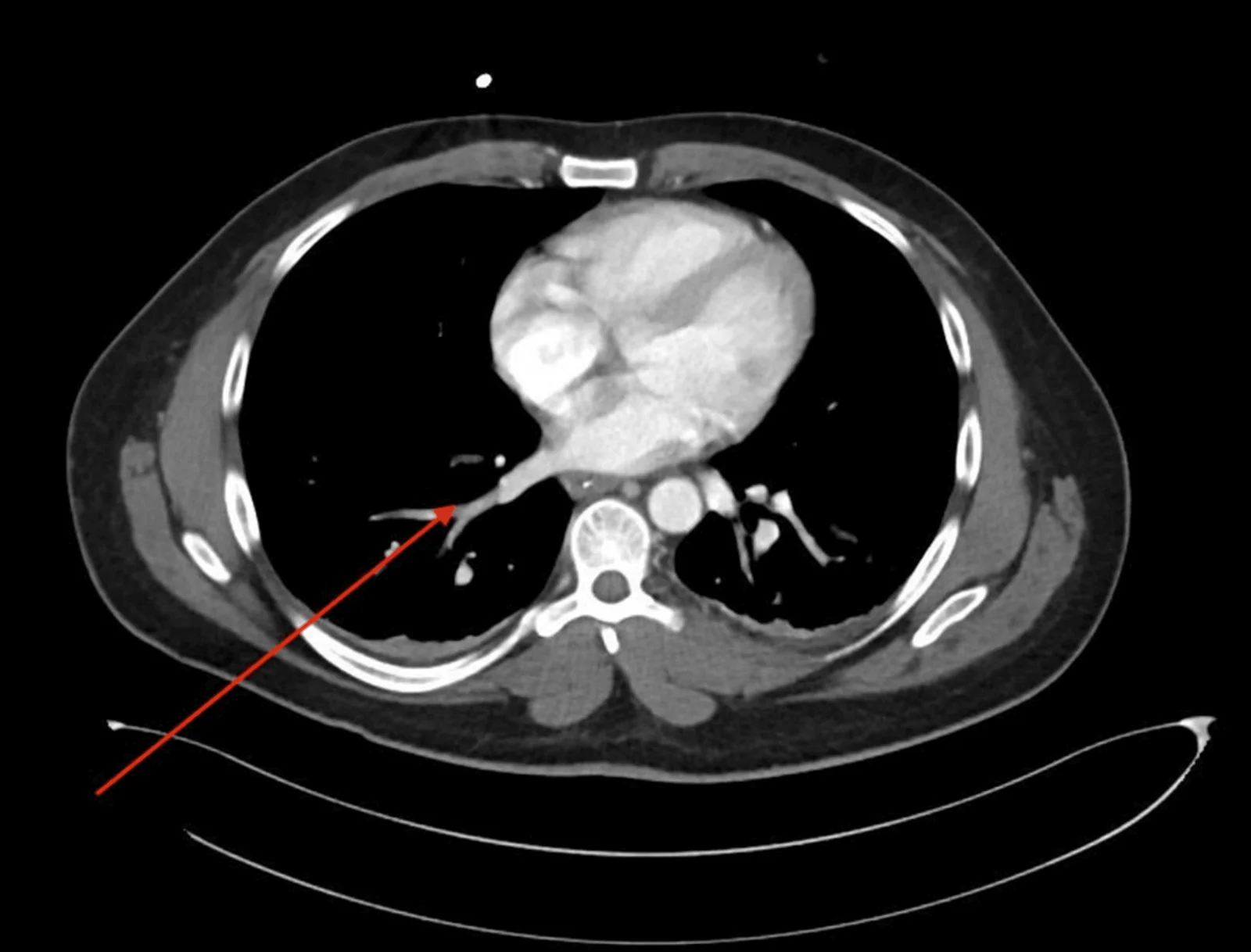
Contrast-enhanced computed tomography pulmonary angiogram (axial view) showing a filling defect in a right sub-segmental pulmonary artery branch, consistent with sub-segmental pulmonary embolism.

Upon arrival to the PACU, the patient was tachycardic but hemodynamically stable, with his elevated heart rate attributed to his perioperative course and blood loss. He remained in the PACU beyond the routine recovery period (defined as a stay exceeding the typical one-to-three-hour recovery window, extending overnight in this case) because no HDU bed was available. Mechanical VTE prophylaxis with sequential compression devices was continued postoperatively. On postoperative day one (POD 1), he developed new tachycardia (122-140 beats/min) and tachypnea (22-24 breaths/min) with nonspecific upper abdominal and lower chest discomfort despite patient-controlled analgesia with fentanyl. His hemoglobin fell from 11.3 g/dL in the immediate postoperative period (POD 0) to 7.1 g/dL on POD 1. He was started on high-flow nasal cannula oxygen (50 L/min) and transfused two units of pRBCs. There was no evidence of active bleeding from the abdominal incision. Computed tomography (CT) of the abdomen showed no intra-abdominal bleeding, while CT pulmonary angiography revealed a right sub-segmental PE (Figure 1).

**Figure 2:**
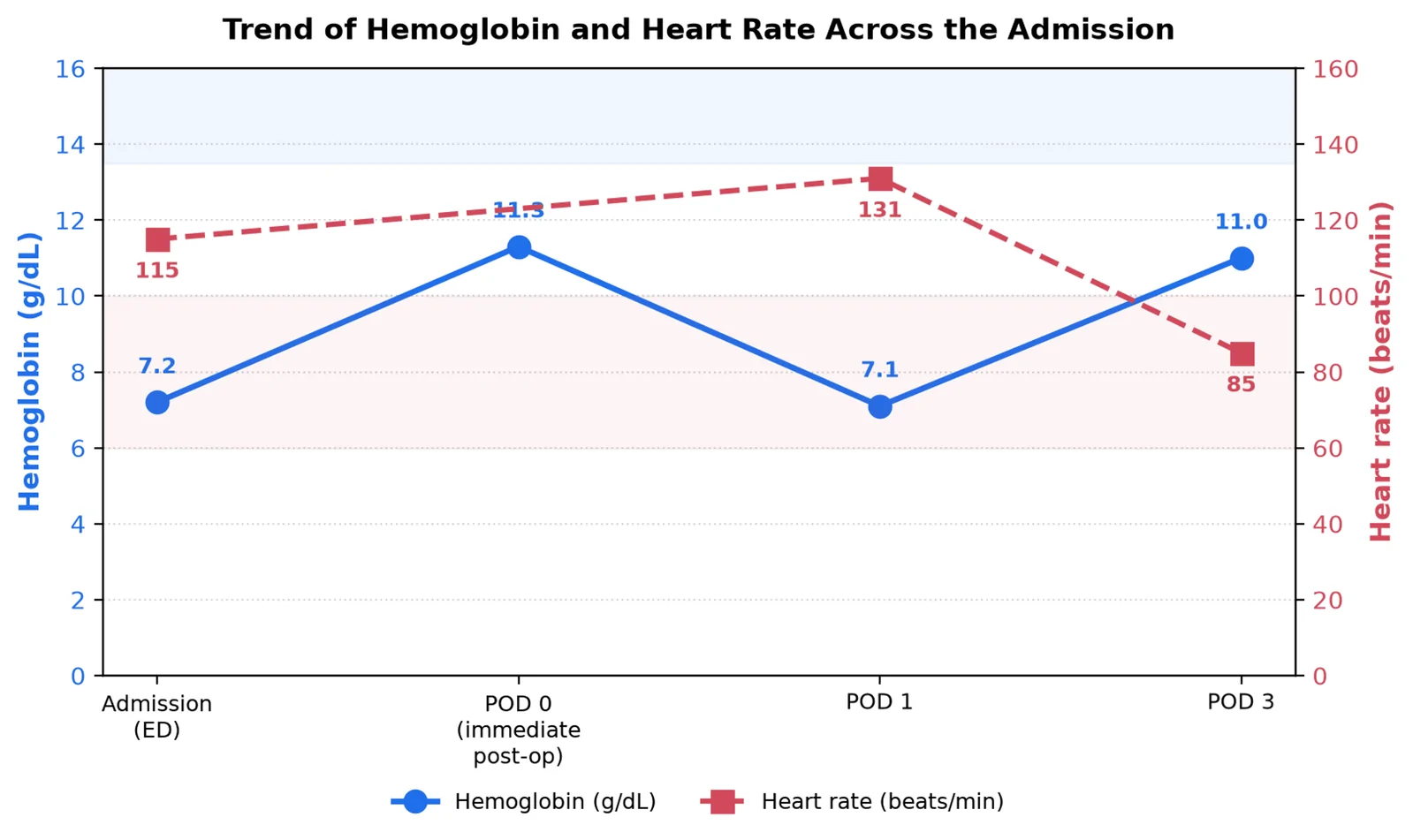
Trend of hemoglobin (blue, left axis) and heart rate (red, right axis) across the admission. The curve demonstrates the abrupt fall in hemoglobin from 11.3 g/dL (POD 0) to 7.1 g/dL (POD 1) coinciding with new tachycardia, and the subsequent normalization of both parameters by POD 3. Shaded bands indicate the respective reference ranges. ED: emergency department; POD: postoperative day

The temporal trend of these parameters, illustrating the abrupt hemoglobin decline and concurrent tachycardia on POD 1 with subsequent normalization by POD 3, is shown in Figure 2.

**Figure 3:**
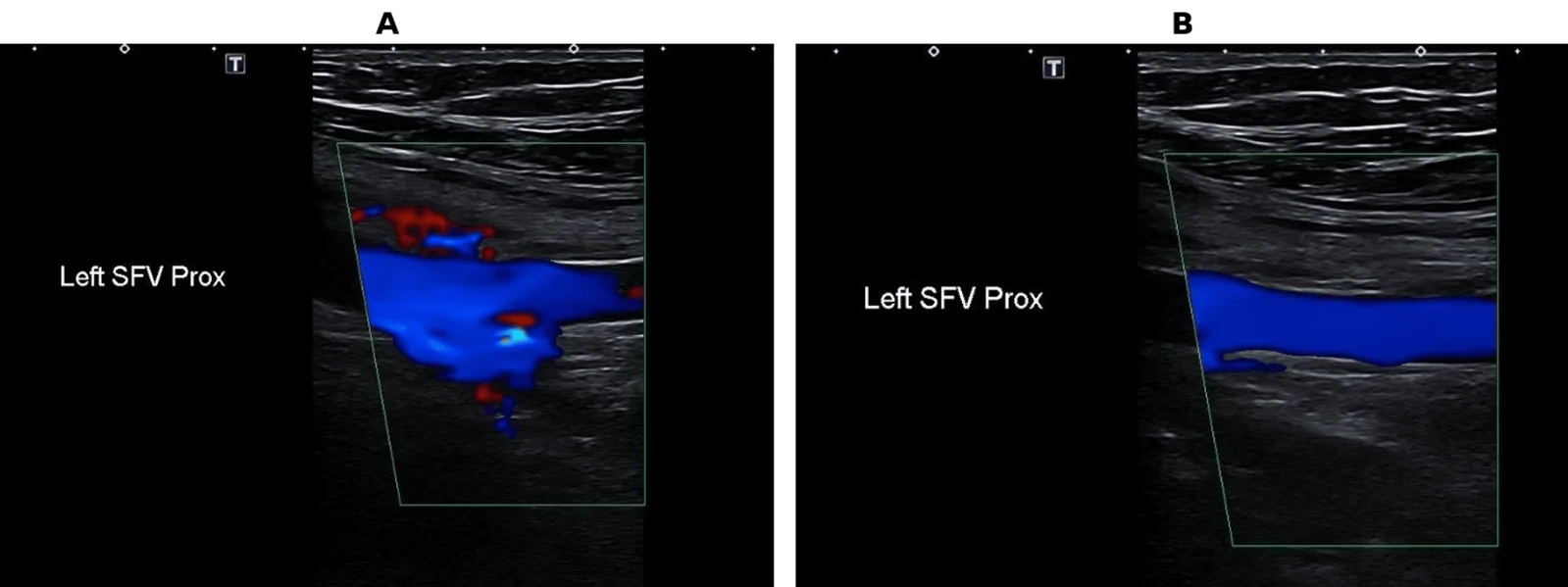
Doppler ultrasound of the left proximal superficial femoral vein (SFV). (A) Color Doppler showing a filling defect with markedly reduced color flow within the vein lumen. (B) Grayscale image with color overlay showing an echogenic intraluminal thrombus with partial flow around it. Both findings are consistent with acute deep venous thrombosis.

He was started on enoxaparin 70 mg (approximately 1 mg/kg) twice daily - a weight-based therapeutic dose consistent with standard guidelines for treatment of acute VTE - and transferred to the surgical ICU. As no preoperative lower-limb Doppler ultrasound had been performed (the patient’s emergency presentation with acute hemorrhage and hemodynamic instability precluded preoperative vascular imaging), Doppler ultrasound of the lower extremities was performed on POD 1, which revealed a fresh DVT in the left superficial femoral vein (Figure 3). The thrombus was described as acute on ultrasound, and the patient had no prior history of DVT, venous insufficiency, or limb swelling, supporting a postoperative origin.

Over the following two days, he was weaned from high-flow oxygen, his heart rate normalized, and his hemoglobin stabilized. His abdominal condition improved, and he progressed from oral fluids to a regular diet. He was transferred to the HDU and discharged home on day five on therapeutic enoxaparin for a planned three-month course. Thrombophilia work-up was unremarkable. At outpatient follow-up, he was well and had returned to work.

## Discussion

The PACU concept originated in 1923 to provide immediate postoperative monitoring [1,7]. While most PACU stays proceed uneventfully, an extended stay both increases the cost of care and exposes patients to additional risk [8,9]. DVT and PE are well-recognized and not uncommon complications of the postoperative period across all care settings, including the surgical ward, HDU, and ICU. The incidence of postoperative VTE is influenced by the type and duration of surgery, patient comorbidities, immobility, and the use (or omission) of prophylaxis. However, PE occurring specifically during an extended PACU stay - as a distinct care environment - has been rarely reported in the literature, with only one prior case described [5].

The extended PACU environment provides a level of monitoring and care comparable to intermediate-level or ICU care, and shares similar thromboembolic risk factors with other postoperative care settings. The postoperative period is inherently proinflammatory and procoagulant because of surgical vascular injury, and the immobility associated with an extended PACU stay compounds this risk, producing a multifold increase in VTE [10]. In this respect, the thromboembolic risk during an extended PACU stay mirrors that of prolonged bed rest in a ward or ICU setting, where immobility, venous stasis, and the postoperative prothrombotic state converge. The extended PACU stay should therefore be viewed not as a unique risk environment, but as one manifestation of the broader postoperative VTE risk that applies across all levels of postoperative care.

To our knowledge, only one prior case of PE arising specifically during a PACU stay has been reported [5]. In that report, Smith and Murauski described a patient who developed PE in the PACU; however, their case differed from ours in several important respects. In the prior case, the PE was identified chiefly on the basis of classic respiratory deterioration, whereas the decisive clue in our patient was the combination of new tachycardia, tachypnea, and - critically - an unexplained hemoglobin drop that could not be attributed to surgical bleeding and that directed attention toward cross-sectional imaging. Compared with that earlier case, our management benefited from an immediate, structured diagnostic pathway: the unexplained hemoglobin decline prompted same-day CT of the abdomen (excluding intra-abdominal bleeding) followed directly by CT pulmonary angiography and lower-limb Doppler ultrasound, allowing a single imaging round to establish both the PE and the concurrent DVT and to initiate therapeutic enoxaparin within hours. This contrasts with many reported postoperative PE cases, in which diagnostic delay results from attributing dyspnea and tachycardia to expected postoperative recovery [11]. Our case therefore illustrates that, in the extended-PACU setting specifically, an unexplained hemoglobin drop - rather than overt chest pain or hypoxia - can be the earliest and most actionable signal of PE, and that a low threshold for combined CT angiography and venous Doppler ultrasound can shorten time to treatment.

Diagnosing PE in the postoperative setting is challenging because symptoms such as chest pain and dyspnea overlap with expected postsurgical findings and residual anesthetic effects, and rapid decision-making is complicated by the logistics of transferring an unstable patient for imaging [11]. In our patient, the key clue was the development of new tachycardia and tachypnea on POD 1, representing a significant change from his immediate postoperative baseline, accompanied by an unexplained hemoglobin drop that prompted imaging.

Our patient had several recognized risk factors for postoperative VTE: emergency surgery, prolonged operative time (five hours), intraoperative blood transfusion, and immobility during the extended PACU stay. Intraoperative blood transfusion has independently been associated with increased postoperative PE risk [12]. Notably, pharmacological VTE prophylaxis was withheld in the immediate perioperative period due to the patient’s active bleeding and recent surgery, a common clinical dilemma that may have contributed to his thromboembolic risk. Mechanical prophylaxis with intermittent pneumatic compression and sequential compression devices was used, though this does not fully eliminate VTE risk, particularly in high-risk patients.

Anticoagulation for PE in the immediate postoperative period is a double-edged sword given the risk of surgical-site or systemic bleeding [13]. Because our patient was young, had no comorbidities or renal impairment, and active bleeding had been excluded, we were able to use therapeutic-dose low-molecular-weight heparin (LMWH) effectively and safely.

It is important to acknowledge that no preoperative Doppler ultrasound was performed in our patient, as his emergency presentation precluded preoperative vascular imaging. While we cannot definitively exclude a pre-existing thrombus, several factors support an acute postoperative origin: the Doppler ultrasound described the thrombus as fresh/acute, the patient had no prior history of DVT or venous insufficiency, he was previously healthy and active, and the temporal relationship with new symptoms on POD 1 is consistent with an acute postoperative event.

## Conclusions

This case highlights that DVT and PE can occur even during a prolonged PACU stay following bleeding or transfusion-heavy surgery. CT pulmonary angiography confirmed PE, while duplex ultrasound identified the source DVT - imaging findings that were essential in guiding urgent anticoagulation and averting further deterioration. Clinicians should maintain a high index of suspicion for VTE in postoperative patients with prolonged immobility, especially when new tachycardia, tachypnea, or unexplained hemoglobin drop occurs, and pursue prompt cross-sectional imaging to confirm diagnosis and guide therapeutic LMWH once bleeding is excluded.
